# Effects of Hot Pepper Waste Powder on Meat Quail Performance, Carcass Yield, and Small Intestine Microflora

**DOI:** 10.1002/vms3.70374

**Published:** 2025-05-05

**Authors:** Olgay Kaan Tekin, Ayşe Gül Filik, Gökhan Filik

**Affiliations:** ^1^ Agricultural Biotechnology Department Agriculture Faculty Kırşehir Ahi Evran University Merkez‐Kırşehir Türkiye

**Keywords:** hot pepper waste powder, microflora, performance, quail, sustainability, waste recycling

## Abstract

In sustainable animal husbandry, wastes generated as a result of agricultural activities need to be evaluated for more effective animal feeding. The world generates approximately 1.2 billion tons of agricultural waste annually. This study aimed to investigate the impact of adding hot pepper waste powder (HPWP) to Japanese quail diets on performance, carcass characteristics, and small intestinal microflora. Each group consisted of 4 replications, with each replication consisting of 10 chicks. A total of 160‐day‐old meat Japanese quail were used in the study, which lasted for 42 days. The experimental groups: included a control group fed with standard feed and groups that had 100, 200, and 400 ppm HPWP added to the ration. At the end of the study, the average live body weights were determined as 275.39, 276.91, 276.15, and 285.92 g, respectively. The average feed intake in the last week was determined as 887.13, 907.33, 890.47, and 936.29 g, respectively. There was a significant increase in feed intake (*p* < 0.01–0.05) in all groups, especially during the growth period (at 2–5 weeks). As a result, while feed consumption and body weight increased in the HPWP added groups compared to the control, there was also a decrease in feed conversion. The study found statistically significant differences in cooking loss, breast L*, b*, chroma, and h° (*p* < 0.01), as well as leg L* and a* values of quail meat with the addition of HPWP (*p* < 0.05). The total bacterial count was 1.5, 15, 1.5, and 1.0 log10^6^ cfu/g, respectively.

## Graphical abstract

Hot pepper waste powder is expected to increase the performance of animals, especially due to its metabolism‐accelerating effect. Broiler quails are among the farm animals expected to gain the most live weight. For this reason, the current study focused on the changes that different doses of hot pepper waste powder could cause in quails.

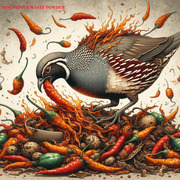

## 1 Introduction

Changes in food consumption habits are influenced by advancements in science and technology, as well as by people's economic status and socio‐cultural levels. The shift in consumer habits emphasises the importance of sustainable food production and consumption. It is crucial to properly utilise all parts of fruits and vegetables that are produced, rather than allowing them to go to waste. Disposing of food waste presents a significant challenge, as the world generates approximately 1.2 billion tons of agricultural waste annually (Tekin 2018). In Türkiye, the annual production of agricultural waste is estimated to be between 50 and 60 million tons. While much of this waste is biodegradable, its improper disposal leads to environmental issues such as water pollution and unpleasant odours. Research into alternative uses for waste parts of fruits and vegetables is growing, and these waste parts are increasingly being utilised in food production.

**Summary**

- To ensure the recycling of vegetable waste.
- To determine the effect of hot pepper waste powder on the performance of broiler quails.
- To determine the feasibility of hot pepper waste powder as a feed additive.

Pepper is a popular type of vegetable that is widely consumed worldwide, including in Türkiye. In addition to being consumed fresh, pepper is used in various forms such as ground pepper, tomato paste, roasting, sauce, pickles, and main dishes. 100 g of fresh pepper contains 29 calories, 1.1 g of protein, 0.2 g of crude fat, 92.6 g of water, 4.2 g of carbohydrates, and 1.4 g of crude cellulose (Keleş 2007; Arabacı 2015). It is also rich in vitamins A, B_1_, B_2_, and C, as well as P (Bioflavonoid) and K vitamins and alkaloids. The crude oil rate in pepper seeds is 25%–28%. Pepper is known to strengthen the proventriculus, increase digestion, and has an appetising effect. Vitamin P in pepper stimulates blood circulation and regulates blood pressure, while vitamin K stops bleeding. It contains bioactive compounds from carotonoids and capsanoids. Capsaicin and capsorubin, β‐carotene, β cryptoxanthin, lutein, zeaxanthin, violaxanthin and antheraxanthin are the most abundant carotenoids and are powerful antioxidants (Shakir 2021). Capsaicins are agonists, and act on transient receptor potential vanilloid channel 1 (TRPV1) receptors in many tissues (Bishnoi et al. 2018; Panchal et al. 2018). These effects include browning of adipocytes, activation of AMP‐activated protein kinase, peroxisome proliferator‐activated receptor, modulation of uncoupling protein 1 and glucagon‐like peptide 1. It has been shown that modulation with capsaicin on these pathways can increase thermogenesis and fat oxidation, improve insulin sensitivity, reduce body fat and improve organ functions (Bishnoi et al. 2018; Panchal et al. 2018; Irandoost et al. 2021). Mechanisms of action of capsaicin and dihydrocapsaicin such as TRPV‐1 gene, peroxisome polyliferator activated receptor, Cannabinoid receptor‐1, Glucagon‐like peptide‐1, protein 1, AMP‐activated protein kinase (AMPK) and adipocytes have been reported (Yasin et al. 2023). Additionally, hot pepper is believed to have an aphrodisiac effect, increasing sexual desire in both men and women. The waste parts of produced pepper can be utilised in various ways. Fruit and vegetable wastes are used in animal nutrition as feed raw materials, contributing to the development of animal products due to their cellulose source and antioxidant properties. Animal protein sources, such as milk, eggs, poultry, ruminant meat, and fish, are important for the physical growth and mental development of children and young people (Gençoğlu et al. 2011). Chicken and quail meat, in particular, are preferred due to their affordability, production ease, low cholesterol, low calorie and low‐fat content, high protein, and calcium amounts. Moreover, chicken and quail meat contain all the essential amino acids required by the human body at adequate levels. They also have a high biological value and are easily digestible. Additionally, their production cost and price are low, making them stand out (Gençoğlu et al. 2011; Cinli 2013). The changes in the food industry have impacted poultry meat production, a category within animal meat production. Changes in quality standards, including aroma, taste, odour, and nutritional characteristics, have influenced people's consumption habits of food products (Yetişir et al. 2008).

In the field of poultry meat production, ongoing research is conducted to understand consumer attitudes and behaviours towards food consumption, and to explore alternative options for consumers. In recent years, there have been studies on the potential use of waste in animal nutrition, with the aim of converting waste materials into food components (Ku and Mun 2008). Research on using the remaining waste from fruits and vegetables as a new, alternative, inexpensive source of protein is ongoing (Dhamankar et al. 1988; Liadakis et al. 1995; Arogba 1997; Moure et al. 2002; Quanhang and Caili 2005; Wani et al. 2006; Boğa et al. 2017; Filik and Kutlu 2018; Filik et al. 2020a; Filik et al. 2020b). This study aimed to determine the effects of adding hot pepper waste powder to quail feed on quail performance, carcass characteristics, and microflora.

## 2 Materials and Methods

### 2.1 Animals and Experimental Design

The present study was prepared as described by Filik et al. (2020a). A total of 160 one‐day‐old Japanese quail chicks were used. The study involved four groups: (1) basal diet without supplementation (0 ppm HPWP: Hot Pepper Waste Powder), (2) basal diet + 100 ppm HPWP, (3) basal diet + 200 ppm HPWP, and (4) basal diet + 400 ppm HPWP. Each group was further divided into 4 subgroups consisting of 10 mixed‐sex chicks, and these subgroups were placed in 5 cages with 2 birds each. The birds were housed in standard cages measuring 40 × 90 × 40 cm, with food and fresh water provided freely. Throughout the study, the chicks were fed starter, grower, or finisher basal diets. The dietary content varied between 23.32% and 19.5% Crude Protein (CP) and 3000–3200 metabolizable energy kg/diet (as shown in Table 1). The basal diets for the quails were prepared by a local company following the NRC (1994) recommendations. In the trial, different groups of quails were given HPWP at doses of 0, 100, 200, or 400 ppm for 7 weeks. The quails were exposed to 23 h L /1 h D of lighting throughout the experiment. The red‐hot pepper used in the study was obtained from a private tomato paste factory in Şanlıurfa, Türkiye (refer to Table 1).

**TABLE 1:** Composition of the starter, grower, and finisher diet (kg/t) (ground form), nutrient content of hot pepper waste powder.

Ingredients	Starter diet (0 to 10 days)	Grower diet (11 to 24 days)	Finisher diet (25 to 42 days)
Maize (7.5% CP)	467.101	544.228	584.749
Soybean meal (46% CP)	387.893	366.087	320.522
Sunflower seed meal (36% CP)	40.000	—	—
Soybean oil	59.803	49.446	59.944
DL‐methionine (99%)	3.530	3.016	2.712
NaCl	2.640	2.313	2.873
Limestone	11.817	8.477	7.595
Dicalcium phosphate (18%)	20.269	18.300	16.252
Vitamin premix*	2.000	2.000	2.000
Mineral premix**	1.000	1.000	1.000
L‐lysine HCl	2.233	3.164	3.164
L‐threonine	0.944	0.598	0.331
Sodium sulphate	0.771	1.371	0.680
**Total (kg)**	**1000.00**	**1000.00**	**1000.00**
Calculated analysis			
Dry matter (%)	88.000	87.681	87.667
Crude protein (%)	23.323	21.500	19.500
Metabolizable energy (kcal/kg)	3000.00	3100.00	3200.00
Crude fibre (%)	4.322	3.686	3.487
Ether extract (%)	8.323	7.519	8.644
Crude ash (%)	6.419	5.684	5.215
Ca (%)	1.049	0.870	0.780
P (%)	0.480	0.435	0.390
Lysine (digestible) (%)	1.280	1.269	1.020
Methionine (digestible) (%)	0.659	0.580	0.530

Nutrient content of hot pepper waste			
Contents	Amount (%)	Contents	Amount (%)
Dry matter	91.63	Crude fibre	30.82
Crude protein	16.35	Acid detergent fibre	62.24
Crude ash	8.82	Neutral detergent fibre	72.05
Crude oil	11.28		

*,**Premix provided per kg of diet: Vitamin A, 12,000 IU; Vitamin D3, 2400 IU; Vitamin E, 30 mg; Vitamin K3, 4 mg; Vitamin B1, 3 mg; Vitamin B2, 7 mg; Vitamin B6, 5 mg; Vitamin B12, 15 µg; niacin, 25 mg; # Fe, 80 mg; folic acid, 1 mg; pantothenic acid, 10 mg; biotin, 45 mg; choline, 125,000 mg; Cu, 5 mg; Mn, 80 mg; Zn, 60 mg; Se, 150 µg.

The hot pepper waste used in the study was powdered before adding to the rations. Feed analysis were performed at University of Kırşehir Ahi Evran, Faculty of Agriculture, Department of Agricultural Biotechnology, Feed Biotechnology Laboratory. The dry matter (DM method 925.40), crude protein (CP: method 984.13), ether extract (EE: method 920.39), Crude ash (CA: method 942.05 [4.1.10]) contents of the samples were determined according to the AOAC procedures (1998). The crude fibre (CF), acid detergent fibre (ADF), and neutral detergent fibre (NDF) were determined according to the Ankom procedures (Ankom Technology 2017a, 2017b, c).

The hot pepper waste used in the study was powdered before adding to the quails' feed. Feed analysis was conducted at University of Kırşehir Ahi Evran, Faculty of Agriculture, Department of Agricultural Biotechnology, Feed Biotechnology Laboratory. The dry matter (DM), crude protein (CP), ether extract (EE), and crude ash (CA contents of the samples were determined according to the AOAC procedures (1998). The crude fibre (CF), acid detergent fibre (ADF), and neutral detergent fibre (NDF) were determined according to the Ankom procedures (Ankom Technology 2017a, 2017b, c).

### 2.2 Analysis

The animals' live weights were measured on the 42^th^ day. Eight animals closest to the average of repetitions were determined in terms of live weight from each replication and were sacrificed by the cut‐to‐length method (Anonymous 1989). Before slaughter, the animals were starved for 12 h to empty their digestive tracts. First, the animals were plucked, and their heads and feet were separated. After the removal of internal organs and abdominal fat, the carcasses, internal organs, and abdominal fat were weighed. The weights and lengths (duodenum, jejunum and ileum) of the intestines were then measured. The hot carcasses and slaughter pieces (legs, breasts, and wings) were also weighed after slaughter. The slaughtered chickens were kept at +4°C for 24 h and their cold carcass weights were determined. Meat from the legs and breasts of eight animals from each replication was analysed for nutrients according to AOAC (1998) standards. The texture, colour, fat content, water holding capacity, and cooking loss of the chicken meat were determined using a texture analyser (CT3 Texture Analyzer), colour analyser (Konica‐Minolta, CR‐410), fat content analyser (Ankom, XT10), and other methods (Şen et al. 2020). Colour analysis of meat was performed with (Konica‐Minolta, CR‐410). L*, a* and b* values ​​were determined. Using these values, Chroma (calculation: square root (a*a) + b*b) and h index (calculation: Degree (chroma value)) were calculated. Colour analysis was performed using a Konica‐Minolta CR‐410, and L*, a*, and b* values were determined. Samples were placed on a white background during measurement (illumination: 0, viewing angle: 90°, measurement aperture diameter: 5 cm). The device was positioned vertically at 90° angle. Thanks to the apparatus of the device, the shutter button was pressed in a light‐proof environment and the colour measurement was completed. From these values, Chroma (calculated as the square root of (a*a) + b*b) and the h index (calculated as the degree of the chroma value) were determined. Microbiological analyses were conducted following FAO (2013) procedures. The total bacterial count (TBC) was determined using plate count agar (Merck, 1.05463, Darmstadt, Germany) at 30°C for 48 h (Mahgoub et al. 2019). Lactobacillus was incubated anaerobically using De Man, Rogosa, and Sharpe (MRS) agar at 37°C for 48 h (Cao et al. 2013). Levene's test for equality of variance was used to analyse the data (Montgomery 2008).

### 2.3 Statistical Analysis

The data obtained from the experiment were subjected to analysis of variance with the General Linear Model (PROC GLM) procedure in accordance with the trial model (Random Plot Trial Plan) using the SAS (1996) package program. Linear, quadratic and cubic effects were determined by orthogonal polynomial contrast (Düzgüneş et al. 1987). The effect of doses of hot pepper waste powder on all measured parameters (treatment) was determined by regression analysis (Düzgüneş et al. 1987). The difference between the groups was made using the Duncan’ Multiple Comparison Method (Genç and Soysal 2018).

## 3 Results and Discussion

The initial live weight, weekly live weight, feed intake, feed conversion ratio of the control and experimental groups fed with HPWP were given in Table 2. At the end of the experiment, there was no statistically significant difference in live weight between the groups. At the beginning of the experiment, the live weights were 9.95 g in the control group, 9.96 g in the 100 ppm HPWP group, 9.98 g in the 200 ppm HPWP group, and 10.00 g in the 400 ppm HPWP group. By the end of the experiment, the live weights of the groups were determined as 275.39 g in the control group, 276.91 g in the 100 ppm HPWP group, 276.15 g in the 200 ppm HPWP group and 285.92 g in the 400 ppm HPWP group, respectively (*p* > 0.05). Reda et al. (2020) found that adding red pepper oil to the diet at different rates (0.75%, 1.25%, 2.25%) increased the performance of quails, but their body weight averages were lower than those determined in the 5^th^ week of our study. On the contrary, Gül and Cufadar (2023) determined that the performance of quails to which they added 300, 600, 900, 1200 mg/kg red pepper oil to their rations in a 5‐week study decreased compared to the control group. Compared to the results of the current study, their live weights correspond to their live weights at the 4^th^ week. Abd EL‐Haliem (2019) adding 1, 2, 3, and 4 per thousand red hot pepper to quail rations did not change the performance of the animals compared to the control group. Current performance data were from the 3^rd^–4^th^ phase of our study. According to these results, HPWP appears to have a performance‐enhancing effect in animals. There were significant differences in feed intake between the groups in the 2^nd^ (*p* < 0.01), 3^rd^ (*p* < 0.05), 4^th^ (*p* < 0.01), and 5^th^ (*p* < 0.05) weeks. The feed intake for each group in grams was as follows: 2^nd^ week–120.31, 130.34, 128.77, and 131.77; 3^rd^ week–244.26, 258.19, 252.01, and 265.84; 4^th^ week–426.22, 448.77, 435.43, and 468.30; 5^th^ week–641.89, 665.64, 651.72, and 690.32. It can be speculated that the importance of feed intake between the groups in the 2^nd^, 3^rd^, 4^th^, and 5^th^ weeks is due to the appetite‐stimulating feature of hot pepper (El‐Deek et al. 2012). The browning of adipocytes due to the selective effects of capsaicin and capsanoids on TRPV1 can be explained by the activation of AMP (adenosine mono‐phosphate)‐activated protein kinase, peroxisome proliferator‐activated receptor, modulated protein 1 and glucagon‐like peptide 1. It has been reported that modulation of these pathways by capsaicin can increase thermogenesis and fat oxidation. In addition, capsaicin may improve insulin sensitivity, reduce body fat and improve organ functions (Bishnoi et al. 2018; Panchal et al. 2018; Irandoost et al. 2021). The feed conversion ratio decreases as the weeks progress due to the fast metabolism of quails and the appetite‐enhancing effect of HPWP, which increase feed intake in groups. However, the group receiving 400 ppm showed improved feed utilisation in the last week compared to the other groups. In a study, it was found that adding 0.05%, 0.10% and 0.15% red hot pepper to the diet increased the feed conversion ratio (Al‐Harthi 2002).

**TABLE 2:** Effects of hot pepper waste powder (HPWP) on the performance of quails.

Parameters	HPWP (ppm)				*p*	Effects^¥^		
	0	100	200	400		*L*	*C*	*Q*
IBW	9.95 ± 0.07	9.96 ± 0.08	9.98 ± 0.07	10.00 ± 0.08	0.962	0.596	0.993	0.997
WLWG^1^	18.68 ± 1.94	21.45 ± 0.10	21.38 ± 0.08	21.37 ± 0.10	0.144	0.077	0.509	0.164
WLWG^2^	60.84 ± 3.29	61.34 ± 2.34	65.27 ± 1.20	63.24 ± 2.34	0.559	0.310	0.604	0.390
WLWG^3^	120.21 ± 3.81	120.44 ± 3.01	120.13 ± 1.75	126.99 ± 4.09	0.388	0.185	0.605	0.323
WLWG^4^	191.03 ± 3.90	193.39 ± 1.97	191.22 ± 2.98	199.96 ± 3.91	0.208	0.105	0.303	0.340
WLWG^5^	239.09 ± 3.10	241.79 ± 2.01	245.39 ± 3.34	245.27 ± 4.57	0.503	0.154	0.762	0.681
WLWG^6^	275.39 ± 4.31	276.91 ± 3.10	276.15 ± 4.18	285.92 ± 6.01	0.333	0.139	0.531	0.370
FI 1 week	28.93 ± 0.18	28.93 ± 0.18	28.93 ± 0.11	28.93 ± 0.19	1.000	0.995	0.997	1.000
FI 2 week	120.31^B^ ± 3.42	130.34^A^ ± 2.19	128.77^A^ ± 0.19	131.77^A^ ± 2.12	0.007**	0.003	0.126	0.137
FI 3 week	244.26^b^ ± 6.61	258.19^ab^ ± 3.79	252.01^ab^ ± 1.01	265.84^a^ ± 6.91	0.041*	0.017	0.094	0.993
FI 4 week	426.22^B^ ± 9.75	448.77^AB^ ± 4.20	435.43^B^ ± 4.21	468.30^A^ ± 12.81	0.010**	0.007	0.041	0.553
FI 5 week	641.89^b^ ± 12.24	665.64^ab^ ± 5.06	651.72^b^ ± 4.77	690.32^a^ ± 17.44	0.028**	0.014	0.083	0.513
FI 6 week	887.13 ± 12.92	907.33 ± 5.15	890.47 ± 11.24	936.29 ± 23.26	0.094	0.056	0.140	0.390
FCR 1 week	1.79 ± 0.35	1.35 ± 0.01	1.35 ± 0.01	1.35 ± 0.01	0.208	0.102	0.564	0.210
FCR 2 week	2.00 ± 0.06	2.14 ± 0.07	1.98 ± 0.04	2.00 ± 0.08	0.224	0.610	0.046	0.894
FCR 3 week	2.04 ± 0.03	2.15 ± 0.05	2.10 ± 0.04	2.10 ± 0.02	0.166	0.387	0.193	0.097
FCR 4 week	2.23^b^ ± 0.02	2.32^a^ ± 0.04	2.28^ab^ ± 0.03	2.34^a^ ± 0.02	0.052	0.034	0.073	0.579
FCR 5 week	2.68^b^ ± 0.03	2.75^ab^ ± 0.03	2.66^b^ ± 0.04	2.81^a^ ± 0.04	0.016	0.069	0.013	0.236
FCR 6 week	3.23 ± 0.05	3.28 ± 0.03	3.23 ± 0.04	3.28 ± 0.05	0.716	0.611	0.304	0.952

Abbreviations: FCR, feed conversion rate; FI, feed intake; IBW, initial body weight; WLWG, weekly live weight gain.

^¥^ *L*, linear; *Q*, quadratic; *C*, cubic effect.

^a,b,c,d^ Means with the different letter within the same column are significantly different according to the Duncan's test at. Significant differences marked within columns with different superscript capital letters indicate *p* ≤ 0.01; lowercase letters indicate *p* ≤ 0.05.

Conversely, another study by Al‐Kassie et al. (2011) reported that red hot pepper added to the diet reduced the feed conversion ratio. At the end of the experiment, the statistically insignificant averages of feed intake and live body weights indicate that the animals completed their development in the 5^th^ week. The study found that there were no significant differences in live weight, hot carcass, cold carcass, leg, breast, wing, back‐neck, abdominal fat, gizzard, liver, heart, proventriculus, and digestive system weight among all groups of HPWP treatment (*p* > 0.05). However, hot carcass and cold carcass weights were lower in the HPWP added groups compared to the control group (Table 3).

**TABLE 3:** Effects of hot pepper waste powder (HPWP) on quail carcass characteristics.

Parameters	HPWP (ppm)				*p*	Effects^¥^		
	0	100	200	400		*L*	*C*	*Q*
Body weight (g)	339.63^ab^ ± 14.45	292.74^b^ ± 16.06	361.75^a^ ± 9.25	322.75^ab^ ± 0.25	0.056	0.744	0.013	0.754
Hot carcass weight (g)	225.22 ± 7.22	182.75 ± 7.60	223.80 ± 10.67	203.53 ± 10.97	0.086	0.593	0.025	0.298
Cold carcass weight (g)	199.81 ± 9.19	162.50 ± 7.46	184.50 ± 13.67	183.08 ± 11.35	0.174	0.806	0.053	0.398
Leg weight (g)	31.93 ± 2.08	26.65 ± 1.40	32.38 ± 2.75	36.75 ± 1.42	0.096	0.086	0.238	0.073
Breast weight (g)	54.89 ± 5.18	55.84 ± 5.23	50.53 ± 2.67	54.30 ± 4.21	0.842	0.740	0.767	0.483
Wing weight (g)	9.95 ± 1.88	8.44 ± 0.08	9.69 ± 0.54	9.57 ± 0.99	0.780	0.981	0.447	0.561
Back‐neck weight (g)	36.04 ± 3.79	31.13 ± 0.55	37.90 ± 6.46	41.67 ± 2.07	0.399	0.246	0.446	0.328
Abdominal fat weight (g)	25.41 ± 7.74	16.35 ± 9.15	23.62 ± 3.58	13.63 ± 12.20	0.749	0.512	0.438	0.960
Gizzard weight (g)	3.76 ± 0.36	2.85 ± 0.05	4.12 ± 0.76	4.00 ± 0.08	0.278	0.354	0.131	0.403
Liver weight (g)	6.80 ± 3.18	7.50 ± 2.65	8.93 ± 3.33	7.54 ± 1.80	0.955	0.787	0.792	0.728
Heart weight (g)	3.00 ± 0.20	2.43 ± 0.18	2.85 ± 0.22	2.55 ± 0.04	0.217	0.285	0.092	0.488
Proventriculus weight (g)	1.15 ± 0.13	0.99 ± 0.16	1.26 ± 0.13	1.38 ± 0.23	0.493	0.274	0.495	0.445
Gastrointestinal weight (g)	14.41 ± 3.23	12.91 ± 3.39	16.59 ± 3.64	14.78 ± 2.77	0.883	0.761	0.507	0.965
Gastrointestinal length (mm)	68.00 ± 5.00	60.00 ± 3.00	69.00 ± 14.00	75.50 ± 1.50	0.601	0.408	0.598	0.395
Carcass loss weight (g)	25.41 ± 1.97	20.25 ± 0.14	23.60 ± 24.33	20.45 ± 0.37	0.987	0.843	0.797	0.938
Hot carcass yield (%)	19.69 ± 2.30	21.62 ± 3.25	17.18 ± 1.69	19.54 ± 1.02	0.614	0.631	0.961	0.642
Cold carcass yield (%)	17.48 ± 2.28	19.23 ± 2.97	15.27 ± 0.26	17.57 ± 1.06	0.603	0.720	0.972	0.621

^¥^ *L*, linear; *Q*, quadratic; *C*, cubic effect.

^a,b,c,d^Means with the different letter within the same column are significantly different according to the Duncan's test at. Significant differences marked within columns with different superscript capital letters indicate *p* ≤ 0.01; lowercase letters indicate *p* ≤ 0.05.

Yılmaz (1994) and Al‐Harthi (2001) reported that red pepper added to the diet reduced carcass weights, while Al‐Kassie et al. (2011) and Shahverdi et al. (2013) found that red pepper added to the diet increased carcass weights. In another study, Al‐Harthi (2002) found that only 0.1% hot pepper group had higher carcass weight. Leg weight was higher in the 200 and 400 ppm HPWP groups, and breast weight was higher only in the 100 ppm HPWP group, but there was no statistical difference between the groups. In a study by Shahverdi et al. (2013), the use of hot pepper increased leg and breast weights, which contrasts with the findings of the current study. Although liver weight was higher in all HPWP groups than in the control group, there was no statistical significance (*p* > 0.05). Studies using hot pepper have shown mixed results, with some reporting increased liver weights (Al‐Harthi 2002, 2001; Al‐Kassie et al. 2011) and others reporting decreased liver weights (Yılmaz 1994). Abdominal fat weight decreased in the HPWP supplemented groups. This decrease may be due to the fat‐burning properties of hot pepper. However, there was no significant difference in abdominal fat weight between the groups. One study reported similar abdominal fat weight in control groups (Shahverdi et al. 2013), while another study (El‐Deek et al. 2012) reported a decrease in abdominal fat in the group that consumed 3 g/kg of hot pepper. There were studies reporting that the addition of hot pepper reduces heart weight (Al‐Harthi 2001; El‐Deek et al. 2012). Al‐Kassie et al. (2011) determined that 0.25% and 0.75% hot pepper additions increased heart weight. In our study, hot carcass yield results were compared to Reda et al. (2020), although they had lower live weight, higher carcass yield was obtained. These results suggest that the increase in live weight in our study may be due to the birds' tendency to drink more water because of HPWP. It's worth noting that carcass yield was even lower in the treatment groups with increasing doses compared to the control group. However, water intake was not monitored between groups throughout the studies. Gül and Cufadar (2023) found carcass yield data from Reda et al. (2020) and Abd EL‐Haliem (2019) to be consistent with their results. On the contrary, Parvari et al. (2022) study on chilli pepper powder, which they added to the diet at rates of 0.75%, 1.25%, 2.25%, supports our current study. The reason for the increase in weight and length of the digestive system due to the addition of HPWP may be related to the amount of feed consumed. The addition of HPWP showed significant differences in the dry matter and crude protein content of quail meat between the groups (*p* < 0.05). Quail meat was found to have higher dry matter and protein content than the control. The dry matter content was 27.43, 28.26, 27.64 and 27.67 and the protein content was 45.33 g, 43.61 g, 44.82 g and 44.44 g, in the 0, 100, 200 and 400 ppm HPWP addition groups, respectively (Table 4). Another study showed that adding 5 g of red pepper to the diet resulted in lower protein content than the control group (Farooq et al. 2022). The study found statistically significant differences in cooking loss, breast L*, b*, chroma, and h° (*p* < 0.01), as well as leg L* and a* values of quail meat with the addition of HPWP (*p* < 0.05). The L* values for breast were measured as 52.31, 46.23, 44.47, and 45.96 in the control, 100, 200, and 400 ppm HPWP groups, respectively. The a* values for breast were measured as 10.79, 11.58, 11.07 and 10.54 in the control, 100, 200 and 400 ppm HPWP groups, respectively. The b* values for breast were measured as 7.76, 7.03, 5.40 and 5.78 in the control, 100, 200 and 400 ppm HPWP groups, respectively. The chroma values for breast were measured as 74.22, 64.47, 41.25 and 45.68 in the control, 100, 200 and 400 ppm HPWP groups, respectively. The h° index values for breast were measured as 35.76, 31.03, 25.95 and 28.56 in the control, 100, 200 and 400 ppm HPWP groups, respectively. The L* values for leg were measured as 44.35, 43.04, 42.21 and 42.07 in the control, 100, 200 and 400 ppm HPWP groups, respectively. The a* values for breast were measured as 11.26, 12.04, 12.07 and 12.01 in the control, 100, 200 and 400 ppm HPWP groups, respectively. L*, a* and b* values represent light‐darkness, red‐greenness and yellow‐blueness, respectively. A high L* value indicates lightness, a high a* value indicates redness, and a high b* value indicates yellowness. L*, a*, and b* values represent lightness‐darkness, redness‐greenness, and yellowness‐blueness, respectively. A higher L* value indicates higher lightness, a higher a* value indicates more redness, and a higher b* value indicates more yellowness. The addition of HPWP resulted in a darker and lighter red breast colour of the quail meats compared to the control group. Similarly, the quail leg colour of the HPWP groups was darker and more yellow than that of the control group (Table 5). Specifically, cooking loss was determined to be 21.20%, 29.54%, 19.22%, and 20.99% in the 0 ppm, 100 ppm, 200 ppm, and 400 ppm HPWP groups, respectively. The results of the study show a significant difference in water loss between breast and leg meat on the 7^th^ day (*p* < 0.01). Specifically, the water loss of breast meat on the 7^th^ day was 31.20%, 27.12%, 29.32%, and 26.84% in the 0, 100, 200, and 400 ppm HPWP groups, respectively. Meanwhile, leg meat water loss was 19.36%, 15.74%, 14.82%, and 16.00% on the 3^rd^ day and 23.56%, 18.73%, 19.83%, and 17.84% on the 7^th^ day. The addition of HPWP resulted in increased water holding capacity of the meat, but also caused higher water holding loss, impacting meat quality. The addition of HPWP made the quail meat drier and tougher in the experimental groups, as shown in Table 6. The study also examined the impact of HPWP on the microbiology of quail small intestine. The total bacterial count was 1.5, 15, 1.5, and 1.0 log10^6^ cfu/g, respectively. The yeast count was determined as 52, 1.7, 1.6, and 66 log10^6^ cfu/g, respectively. The count of aerobic lactic acid bacteria was 0.5, 2.4, 0.1, and 1.8 log10^5^ cfu/g, respectively. The count of anaerobic lactic acid bacteria was 2.1, 0.8, 0.8, and 2.5 log10^6^ cfu/g, respectively.

**TABLE 4:** Effects of hot pepper waste powder (HPWP) on chemical quality of quail meat.

Parameters	HPWP (ppm)				*p*	Effects^¥^		
	0	100	200	400		*L*	*C*	*Q*
DM (g)	27.43^b^ ± 0.16	28.26^a^ ± 0.22	27.64^b^ ± 0.19	27.67^b^ ± 0.16	0.018	0.893	0.011	0.029
CP (g)	45.33^a^ ± 0.29	43.61^b^ ± 0.53	44.82^ab^ ± 0.38	44.44^ab^ ± 0.42	0.031	0.434	0.017	0.108
Crude fat (g)	28.62 ± 0.42	29.47 ± 0.38	29.46 ± 0.57	28.48 ± 0.56	0.317	0.846	0.957	0.063
Crude ash (g)	8.80^b^ ± 0.17	9.84^a^ ± 0.33	9.53^ab^ ± 0.24	9.43^ab^ ± 0.28	0.051	0.188	0.192	0.037
Crude protein, DM%	12.40 ± 0.09	12.24 ± 0.17	12.44 ± 0.12	12.32 ± 0.12	0.709	0.954	0.245	0.898
Crude fat, DM%	7.87 ± 0.10	8.31 ± 0.11	8.16 ± 0.19	7.97 ± 0.11	0.088	0.774	0.365	0.018
Crude ash, DM%	2.43^B^ ± 0.05	2.81^A^ ± 0.08	2.67^AB^ ± 0.07	2.62^AB^ ± 0.07	0.003	0.179	0.060	0.003

^¥^ *L*, linear; *Q*, quadratic; *C*, cubic effect.

^a,b,c,d^Means with the different letter within the same column are significantly different according to the Duncan's test at. Significant differences marked within columns with different superscript capital letters indicate *p* ≤ 0.01; lowercase letters indicate *p* ≤ 0.05.

**TABLE 5:** Effects of hot pepper waste powder (HPWP) on the physical quality of quail meat.

Parameters	HPWP (ppm)				*p*	Effects^¥^		
	0	100	200	400		*L*	*C*	*Q*
WHC, %	16.54 ± 0.89	17.43 ± 0.78	19.05 ± 1.06	18.36 ± 0.94	0.245	0.089	0.464	0.392
Freezing loses, %	1.85 ± 0.10	1.65 ± 0.12	1.82 ± 0.19	1.92 ± 0.15	0.587	0.556	0.486	0.298
Cooking loses, %	21.20^B^ ± 2.46	29.54^A^ ± 2.28	19.22^B^ ± 1.61	20.99^B^ ± 1.58	0.003	0.220	0.001	0.104
Shear force (cm^2^/g)	958.48 ± 32.09	1062.34 ± 38.53	999.27 ± 43.27	998.54 ± 38.76	0.301	0.741	0.176	0.184
Breast L*	52.31^A^ ± 1.18	46.23^B^ ± 0.94	44.47^B^ ± 0.53	45.96^B^ ± 0.71	0.000	0.000	0.785	0.000
Breast a*	10.79^ab^ ± 0.36	11.58^a^ ± 0.31	11.07^ab^ ± 0.21	10.54^b^ ± 0.20	0.056	0.308	0.310	0.019
Breast b*	7.76^A^ ± 0.31	7.03^A^ ± 0.31	5.40^A^ ± 0.17	5.78^B^ ± 0.22	0.000	0.000	0.014	0.036
Breast chroma	74.22^A^ ± 5.19	64.47^A^ ± 4.47	41.25^B^ ± 2.02	45.68^B^ ± 2.84	0.000	0.000	0.018	0.067
Breast h^o^	35.76^A^ ± 1.13	31.03^B^ ± 1.18	25.95^C^ ± 0.64	28.56^BC^ ± 0.86	0.000	0.000	0.069	0.000
Leg L*	44.35^a^ ± 0.53	43.04^ab^ ± 0.68	42.21^b^ ± 0.71	42.07^b^ ± 0.50	0.036	0.006	0.940	0.345
Leg a*	11.26^b^ ± 0.25	12.04^a^ ± 0.19	12.07^a^ ± 0.21	12.01^ab^ ± 0.22	0.026	0.022	0.507	0.057
Leg b*	6.35 ± 0.20	6.46 ± 0.32	6.65 ± 0.34	6.26 ± 0.15	0.748	0.950	0.579	0.343
Leg chroma	53.04 ± 3.13	57.44 ± 5.33	60.31 ± 5.55	52.05 ± 1.99	0.488	0.996	0.616	0.141
Leg h^o^	29.40 ± 0.69	27.84 ± 1.04	28.56 ± 1.15	27.60 ± 0.62	0.493	0.249	0.328	0.740

WHC, water holding capacity.

^¥^ *L*, linear; *Q*, quadratic; *C*, cubic effect.

^a,b,c,d^Means with the different letter within the same column are significantly different according to the Duncan's test at. Significant differences marked within columns with different superscript capital letters indicate *p* ≤ 0.01; lowercase letters indicate *p* ≤ 0.05.

**TABLE 6:** Effects of hot pepper waste powder (HPWP) on water loss in quail meat.

Parameters, %	HPWP (ppm)				*p*	Effects^¥^		
	0	100	200	400		*L*	*C*	*Q*
Breast water loss (3^rd^ day)	26.24 ± 0.70	24.57 ± 0.70	26.48 ± 0.97	24.27 ± 0.78	0.116	0.267	0.031	0.736
Breast water loss (7^th^ day)	31.20^A^ ± 0.84	27.12^BC^ ± 0.79	29.32^AB^ ± 0.97	26.84^C^ ± 0.73	0.001	0.005	0.004	0.344
Leg water loss (3^rd^ day)	19.36^A^ ± 1.18	15.74^B^ ± 0.61	14.82^B^ ± 0.57	16.00^B^ ± 0.93	0.002	0.005	0.871	0.005
Leg water loss (7^th^ day)	23.56^A^ ± 0.90	18.73^B^ ± 0.82	19.83^B^ ± 1.22	17.84^B^ ± 1.25	0.001	0.001	0.056	0.182

^¥^ *L*, linear; *Q*, quadratic; *C*, cubic effect.

^a,b,c,d^ Means with the different letter within the same column are significantly different according to the Duncan's test at. Significant differences marked within columns with different superscript capital letters indicate *p* ≤ 0.01; lowercase letters indicate *p* ≤ 0.05.

In a related study, McElroy et al. (1994) found that 5 ppm capsaicin exhibited resistance against *Salmonella* spp. on the 28^th^ day, while 20 ppm capsaicin showed resistance on the 21^st^ and 42^nd^ days. Jensen et al. (2003) reported that adding 3000 SHU capsaicin to poultry feed reduced *Salmonella* contagion by preventing rodent growth and intake of feed in the poultry house. Puvača et al. (2014) found that adding red‐hot pepper to the ration had an antimicrobial effect (Table 7). Zeweil et al. (2011) discovered that including 1.5% and 0.75% hot pepper in the diet led to a decrease in the numbers of *E. coli*, *Salmonella*, and *Streptococci*. Meanwhile, Alagawany et al. (2020) found that adding a combination of red and black pepper oil to the birds' diet reduced the total number of bacteria, *lactobacilli*, *coliform*, *Salmonella*, and *E. coli*. In a separate study that used red pepper oil, quails that were fed with 0.8, 1.2, and 1.6 g/kg of red pepper oil showed a decrease in total bacterial count, *Lactobacilli, coliform, E. coli*, and *Salmonella* colonisation compared to the control group (Reda et al. 2020). Capsaicin has been reported to be beneficial for the intestinal health of animals (Liu et al. 2021). It has been reported that capsaicin in the diet can reduce *Salmonella enteritidis* in chickens (Vicente et al. 2007) and 1.98% capsaicin can reduce the density of *E. coli* and *Clostridium perfringens* in the rectum (Jamroz et al. 2003). This has been attributed to capsaicin, which has bactericidal activity against intestinal pathogens (Gram‐positive and Gram‐negative) (Omolo et al. 2014; Agarwal et al. 2017; Salem et al. 2021). Some phenylpropanoids of Capsicum species have been suggested to have bacteriostatic activity such as t‐cinnamic, o‐coumaric, m‐coumaric, ferulic acids or bactericidal activity such as caffeic acid (Abdul Aziz 2010). In particular, the increase in microvilli length and circumference increases the absorption surface of the small intestine, thus increasing the bioavailability of nutrients (Srinivasan 2016; Filik et al. 2020a). According to the study results, it was determined that the use of HPWP increased the feed intake, body weight gain, and feed conversion ratio of quails. It is thought that the result may be due to the appetising feature of hot pepper. Additionally, the physical and chemical analyses of quail meat with HPWP added to the ration showed that the meat becomes dry, crispy, and contains less water. It has been observed that when animals are fed with HPWP supplemented feed, they are in constant motion and their water intake needs increase when they consume the feed. As a result of the increase in their metabolism, it can be associated with the decrease in leg weight and increase in breast weight of the animals in the groups given HPWP. In the poultry sector, broiler chickens are sold both whole and in pieces, while quail meat is sold whole. In today's world where the preferences of consumers have changed in general terms, it is predicted that it is possible to sell quail separately as breast meat or thigh meat with these results. Thus, alternative products may emerge for both producers and consumers.

**TABLE 7:** Effects of hot pepper waste powder (HPWP) on quail small intestine microflora.

Microorganisms	HPWP (ppm)				*p*	Effects^¥^		
	0	100	200	400		*L*	*C*	*Q*
Total bacteria (log10^6^ cfu/g)	15 ± 5.0^B^	150 ± 10.0^A^	15 ± 5.0^B^	10 ± 1.0^B^	**0.000**	0.000	0.000	0.000
Yeast (log10^5^ cfu/g)	5.2 ± 0.20^B^	17.5 ± 0.5^A^	16 ± 1.0^A^	7.63 ± 2.93^B^	**0.000**	0.1906	0.1258	0.000
Lactic acid bacteria (aerobic) (log10^4^ cfu/g)	5.2 ± 1.8^C^	24 ± 2.0^A^	1.2 ± 0.20^D^	18 ± 2.0^B^	**0.000**	0.0070	0.000	0.3325
Lactic acid bacteria (anaerobic) (log10^5^ cfu/g)	21 ± 2.0^B^	8.0 ± 2.0^C^	3.4 ± 0.20^D^	25 ± 2.0^A^	**0.000**	0.1372	0.0041	0.000

^¥^ *L*, linear; *Q*, quadratic; *C*, cubic effect.

^a,b,c,d^Means with the different letter within the same column are significantly different according to the Duncan's test at. Significant differences marked within columns with different superscript capital letters indicate *p* ≤ 0.01; lowercase letters indicate *p* ≤ 0.05.

## Author Contributions

**Olgay Kaan Tekin**: Investigation, methodology, writing – review and editing, formal analysis. **Ayşe Gül Filik**: Methodology, writing – review and editing, writing – original draft, formal analysis. **Gökhan Filik**: Supervision, writing – original draft, writing – review and editing, investigation, resources, formal analysis.

## Ethics Statement

The approval of the Local Ethics Committee for Animal Experiments was obtained by decision of the Local Ethics Committee for Animal Experiments of Kırşehir Ahi Evran University dated and numbered 26/07/2016‐09‐01

## Conflicts of Interest

There are no conflicts of interest to declare.

## Data Availability

The original contributions presented in the study can be directed to the corresponding author.
